# Home-delivered meal boxes in a family setting: a qualitative study investigating reasons for use and perceived impact on meal practices

**DOI:** 10.1186/s12889-024-17729-1

**Published:** 2024-01-23

**Authors:** Marjolijn Vos, Benedicte Deforche, Wendy Van Lippevelde

**Affiliations:** 1 Department of Marketing, Innovation and Organization, Faculty of Economics and Business Administration, Ghent University, Ghent, Belgium; 2 Unit Health Promotion, Faculty of Medicine and Health Sciences, Department of Public Health and Primary Care, Ghent University, Ghent, Belgium; 3 Movement and Nutrition for Health and Performance Research Group, Faculty of Physical Education and Physiotherapy, Vrije Universiteit Brussel, Brussel, Belgium

**Keywords:** Family, Children, Home-delivered meal box, Meal practice, Determinants

## Abstract

**Background:**

Cooking and consuming a homemade meal is associated with health benefits. Home-delivered meal boxes can support families in cooking this fresh meal. The current study aimed to gain a deeper understanding of the determinants of meal box use, and of the perceived impact on meal practices of parents with younger (i.e., aged 6–12 years) and older children (i.e., 13–18 years).

**Methods:**

Four focus groups were conducted (*n* = 19); two with parents of younger children, and two with parents of older children. A semi-structured interview guide was developed and interviews were recorded and transcribed. Reflexive thematic analysis was performed using NVivo 1.4.

**Results:**

Most parents mentioned practical reasons like saving time and money, as well as inspiration, as reasons to choose a home-delivered meal box. Also, tastiness and menu variation were often mentioned as determining factors by both parent groups. However, a few parents stated to stop using the meal boxes because of returning menus or too small portion sizes. Meal box providers were chosen based on the price, the freshness and the quality of the products. Moreover, positive effects on parents’ perceived cooking skills and knowledge were reported. Also, some parents mentioned positively changed attitudes towards vegetarian dishes. Lastly, parents reported healthier eating due to more appropriate portion sizes and more vegetables. A prominent difference between parent groups was that older children played a role in continuing the use of meal boxes, and helped to prepare the meals (contrary to younger children).

**Conclusions:**

Home-delivered meal boxes might be promising to enhance families’ meal practices. This study could inform social marketeers and health promotors to adopt an optimal strategy to reach families.

**Supplementary Information:**

The online version contains supplementary material available at 10.1186/s12889-024-17729-1.

## Background

Navigating the food environment to ensure regular healthy food intake, is an ongoing challenge for many consumers [1]. Moreover, with convenience foods (e.g., frozen pizzas, ready-to-eat meals) being omnipresent [2], it is assumed that cooking and cooking skills of the general adult population have declined when they are changing rather than being in decline [3]. Cooking skills are affected by structural changes in the labor market (i.e., more families with two-earners), new cooking technologies and new kinds of diets, which implies changes in the practices and understandings of the whole cooking process, rather than a decline in skill level. Contemporary meal provisioning is characterized by hybridity, on the intersection of convenience-based meals and homemade meals [3]. Home-delivered meal boxes are a great example of a food industry development that is a combination of home-cooking and convenience-based meals. Meal boxes consist of premeasured food items with an accompanying recipe and are delivered to households after ordering online [4].

Cooking a homemade meal is considered to positively influence an individual’s own food intake and meal practices, as well as those of the whole family [5, 6]. Family meals are associated with healthy diet quality [7], as well as with improved psychosocial outcomes in children and adolescents (i.e., reduced risk behaviors and less depressive symptoms) [8, 9]. Parents consider some key characteristics important for defining a family meal: the dinner is homemade, it is prepared by the main caregivers, it is eaten at home at a table/counter, most of the family is gathered, and a conversation free of distractions is occurring [10]. Recently a framework around the family meal was developed by Middleton and colleagues [11]. This Family Meal Framework attempts to describe the cyclical and reactive nature of the work that is put into implementing a family meal. Five main components are described: cognitions, actions, outcomes, beliefs and feelings, and responsibilities. The framework considers the impact that family members’ experiences can have on cognitions and actions that precede the meal (i.e., the component "outcome") [11]. For example, children who are fussy eaters can frustrate parents to the point that they change their meal preparation strategy. According to this framework, a meal box can support both cognitive practices and actions involved to execute a family meal, by for example reducing time and effort (i.e., the cognitive work) [11].

So far, only a few studies looked into the added value of meal boxes on meal practices in a family context and found that they can facilitate active participation of children and adolescents [12–15], as well as enhance health-beneficial behaviors (i.e., preparing a fresh meal) and family bonding time [5, 15]. However, the reasons for parents to start and continue to choose such a meal box have not yet been explored. Moreover, given the previously found positive results of home-delivered meal boxes, they might play a role in influencing parents’ meal practices. To our knowledge, there has been no research investigating a possible difference in perceived impact between families with younger or older children. Thus, we want to explore a possible difference between those families because previous dietary behavior intervention programs targeting younger children have been found to be more successful than those targeting adolescents [16]. With this qualitative study, we want to gain a deeper understanding of determinants of meal box use, and the perceived impact on meal practices among parents with younger (i.e., aged 6–12 years), and older children (i.e., 13–18 years).

## Methods

### Study design

To find out how home-delivered meal boxes affect families, focus group interviews were conducted from February until April 2021, in Flanders, Belgium. Focus groups were used because they allow to build further on every experience or idea that is shared by an individual in a group discussion, without trying to reach a consensus [17]. A qualitative approach is preferred over quantitative research methods because we aim to get a deeper understanding of the determinants of meal box use and the perceived impact [18]. A general inductive approach was used for this study, which implies no prior assumptions or theories, but interpretations made from the raw data [19]. Ethical approval was granted by the Ethical Committee of the Faculty of Economics and Business Administration of Ghent University. The quality of the research was assessed against the Consolidated Criteria for Reporting Qualitative Research checklist to ensure quality of reporting [20].

### Participants and recruitment

To be eligible for participation, individuals had to have at least one child still living at home and have experience with the use of meal boxes. The latter was broadly defined, meaning that participants could have used the meal boxes in the past, or still order them sometimes/frequently. Also, parents who use “fresh packages” from the grocery store (i.e., a meal package with individual fresh products and a short recipe, that is available in many grocery stores) were included. A distinction between families with younger children (6–12 years) and families with older children (13–18 years) was made. To recruit parents, a message was shared via various social media channels. Due to restrictive Covid-19 measures, it was difficult to recruit parents in a "physical way", by for example handing out flyers at schools. The social media message contained the aforementioned inclusion criteria to select participants (i.e., purposive sampling [21]). It should be noted that due to recruiting via social media, two participants were acquaintances of the research assistants. Participants signed an informed consent form prior to participation.

### Data collection

Due to Covid-19 measures, the focus groups were held online via Zoom. The number of participants per focus group discussion was limited to a maximum of six, to ensure that all participants could participate in a well-structured conversation. Data collection was conducted by two research assistants, who received training from the first author (MV, PhD) on how to conduct focus groups. Every focus group was carried out by a moderator who guided the conversation and an assistant who made notes and assisted with technical practicalities. Focus groups lasted no longer than one hour, and were video-recorded and transcribed, allowing for further analysis afterwards. Participants were not contacted for validation of transcripts.

A semi-structured interview guide was developed (see Additional file 1) based on the research question and existing literature. Following an introduction and warm-up exercise, participants were asked why they use meal boxes and how important a healthy lifestyle is for them. Then, key questions were addressed, asking about advantages and disadvantages of meal boxes, whether there is a perceived change in the family’s eating behavior since using meal boxes and which impact children experienced from, or exerted themselves on the choice for meal boxes. The focus groups concluded by summing up main points and thanking the participants. Before the start of the discussion, participants filled in a short sociodemographic questionnaire inquiring about age, sex, number of children and their age, marital status, education, both their occupation and that of their partner, and a few questions on meal box use (see Additional file 2).

### Data analysis

Transcripts of recordings were managed for analysis using NVivo version 1.4. Six phases of reflexive thematic analysis were followed [22] to explore and interpret the data. Inductive reasoning (i.e., codes developed throughout the process of engaging with the data) helped generate themes. Two research assistants analyzed the data independently, ensuring reliability of data synthesis. The research assistants and first author (MV) discussed the process of analysis and agreed that data saturation was reached with the last focus group. Moreover, in a later phase, results were analyzed by MV, compared with the results of the research assistants, and partly analyzed (50%) by the last author (WVL), as well as discussed with WVL and BD. In this last discussion, the fourth step of the thematic analysis was further executed by reviewing if the themes address the research question sufficiently. SPSS Statistics 27 was used to describe the focus group sample and analyze the quantitative data obtained from the questionnaires.

## Results

### Demographics

Table 1 provides a demographic overview of each parent group. For the first focus group, six participants were recruited of which four eventually were present. Considering no-show rates, more participants were recruited in the following three focus groups. A total of 19 parents, in four focus groups, participated in the study. Two focus groups were held with parents with younger children (*n* = 9), and two with parents with children of adolescent age (*n* = 10). Of the 19 participants, there were three who only used fresh packages from the grocery store and no meal boxes. Even though not all questions applied to the situation of those latter participants, it was decided to still include them in this study as some interesting results were found based on their input.

**Table 1:** Demographics

	Parents of younger children (6–12 years)	Parents of older children (13–18 years)
	***% (n) or Range (mean ± SD)***	***% (n) or Range (mean ± SD)***
**Total particpants**	100 (9)	100 (10)
**Age**	35–46 (40 ± 3.1)	42–54 (48 ± 4.1)
**Sex**		
Female	78 (7)	60 (6)
Male	22 (2)	40 (4)
**Number of children** ^**a**^		
2	89 (8)	40 (4)
≥ 3	11 (1)	50 (5)
**Education** ^**a**^		
College	78 (7)	60 (6)
University	11 (1)	40 (4)
**Profession** ^**a**^		
Blue collar employee	/	10 (1)
White collar employee/teaching in lower and secondary education	89 (8)	30 (3)
White collar employee management/teaching in higher education and university	/	50 (5)
No profession	/	10 (1)
**Parent’s diet**		
Omnivore	78 (7)	60 (6)
Flexitarian	22 (2)	30 (3)
Vegetarian	0	10 (1)

^a^ Missing data *n* = 1

Two main themes were developed, namely: (1) determinants of; and (2) perceived impact of home-delivered meal box use. The results will be discussed according to these themes, and for all participants (i.e., for both parent groups).When a difference between families with younger and older children is found, then this is clearly stated.

### Determinants of home-delivered meal box use

#### Characteristics of home-delivered meal boxes

Home-delivered meal boxes were mostly chosen for following reasons: convenience, inspiration, saving money, and tastiness of the dishes. First, almost all participants mentioned a busy life as a reason to start using meal boxes or fresh packages. They stated that a full fridge brings peace of mind and that especially in combination with a job and children, saving time is essential to reduce stress. The fact that meal boxes were delivered at home saved a lot of time, according to most parents who used them. Also, some participants mentioned appreciating the flexibility of the ordering system; many providers do not work with a fixed subscription but allow ordering each week.

*“You come home and take it out of the fridge, you don’t have to visit a store anymore. You don’t lose any time. Also, everything is ready from the garlic clove to the spices you need.” Female, 42 yrs, 6–12 age group*.

*“Yes it gives you much more peace of mind because you always have a filled fridge, you always know what you are going to eat.” Female, 51 yrs, 13–18 age group*.

However, not all participants found cooking meal boxes time-saving, but the fact that they did not have to go grocery shopping compensated them for spending more time preparing the meal. A few participants seemed to enjoy taking their time to cook the dish.

*“For us, the time-saving aspect actually never really played a role because we spend more time on cooking now.” Male, 38 yrs, 6–12 age group*.

*“I think it is worthwhile to spend a bit more time on cooking the recipes, some of which you cook for the first time, in view of the fact that you need to do grocery shopping less frequently.” Male, 54, 13–18 age group*.

Second, most participants were happy that they did not need to think about the week menu and what to cook every day. The question “What do we eat tonight?” became irrelevant. Meal boxes provided inspiration, and participants seemed to appreciate the new recipes and the new flavors they learned this way.

*“Yes, they are creative. I think that’s a big advantage. That for once the thinking is left to someone else. That’s already one ‘must do’ less!” Female, 40 yrs, 6–12 age group*.

Third, money was another factor often mentioned for using meal boxes. It seemed that many participants found ordering meal boxes cheaper than doing all the grocery shopping themselves. According to them, this is because store trips were reduced, as well as the in-store temptation to buy other, maybe not necessary products. Another reason was receiving and paying for adjusted portions, which was not only important to save money, but also to prevent food waste and over-eating. Moreover, parents mentioned wasting less energy (i.e., no transport costs, and losing less time). So, most participants outweighed all aspects of the meal boxes instead of the price alone and then stated that the price-quality ratio is good. One participant, however, thought it was more expensive to buy a meal box because you save money when you choose recipes and buy it yourself.

*“I don’t know by heart how much I’m paying right now, but it’s really not expensive. If I had to pay for it all separately and the transport that you would otherwise have to charge as well…I certainly don’t think it’s more expensive.” Female, 40 yrs, 6–12 age group*.

*“I think if you do your own shopping and decide which recipes you will prepare in advance, that it does get cheaper, doing it yourself. But it doesn’t outweigh the convenience, of course. Plus when you walk in the store you always take things you didn’t plan for.” Female, 52 yrs, 13–18 age group*.

A few users of fresh packages in the grocery store also mentioned advantages of inspiration and saving time. Generally, fresh packages were found to be more traditional dishes, but still seemed to inspire to cook a fresh meal. Also, with adjusted portions (which also reduced food waste) and pre-cut vegetables, time was saved. However, the menu variation was mentioned to be less large than with home-delivered meal boxes.

*“We also regularly go to store X, very consciously to buy boxes to make soup, a soup that we would never think of making ourselves. My husband really likes to prepare a tajine, you can buy everything in one box right away, you still have to buy meat, but you already have the right spices etc.” Female, 48 yrs, 13–18 age group*.

Lastly, an important aspect of meal boxes for most participants, and especially for the children, was the tastiness of the dishes. A few parents with children aged 6 to 12 stopped ordering meal boxes because children did not like them and found them too special (e.g. use of ginger and lentils). However, also some participants reported convincing children to at least try the food, or preparing the meal as such that children do like it. Seemingly, depending on the meal box supplier, dishes were more child-friendly. Most parents in both age categories considered their children’s preferences when they chose the menu (i.e., by knowing what they (dis)liked). It should be mentioned also that a few partners of the parents were not a fan of the meal boxes (because of too many vegetables and too small portion sizes). Apart from tastiness, the variation in the provided meal box menus was a positive point for all participants. However, also here some parents of younger children stopped using meal boxes or switched to a different supplier when after a while the same menu options returned.

*“We often do it without the kids because we find that sometimes it is a little too experimental for them.” Male, 38 yrs, 6–12 age group*.

*“In the beginning it was very limited, now it is much better. Still, you notice that the same spices and basics often come back in the recipes. So after a while I do think you’ve had it.” Female, 46 yrs, 6–12 age group*.

Adolescent children seemed to play a vital role in continuing the use of meal boxes because they wanted to try something different (i.e., variation) or wanted to help prepare the dishes. Many adolescents convinced their parents to buy a meal box and to also consider vegetarian meals (i.e., they helped choose the weekly menu).

*“Here the boys said “shouldn’t we try this” because they also see the commercials right and left.(….) in the beginning I was a bit hesitant, but they managed to convince me.” Female, 51 yrs, 13–18 age group*.

*“I think here the kids are even the main drivers to keep doing it! They choose it. They always choose something for 3 days: 1 pasta dish, 1 vegetarian and 1 meat dish. And also because then they cook along, otherwise they don’t!” Female, 42 yrs, 13–18 age group*.

#### Characteristics of meal box providers

Many participants mentioned comparing various meal box suppliers and looking at different aspects to make their choice. The environmental impact of meal boxes was addressed by both parent groups. Herein food waste, environment-friendly packaging and seasonal products were found to be important. Less food was wasted because portion sizes were tailored to the target group, and because participants engaged in less grocery shopping. However, a few participants mentioned that the portion size was either not enough, or that there were leftovers because they did not cook the meal at all. This was then a reason to stop using meal boxes again.

*“It’s all measured too, isn’t it? If you need 2 chicory stalks for example, there are also only 2 in there and you don’t have to buy the compulsory 4, so I find that really convenient.” Female, 37 yrs, 6–12 age group*.

*“I started provider X out of curiosity, but I switched for sustainability reasons” Female, 45 yrs, 13–18 age group*.

Linked to the seasonal products, a few participants preferred locally-produced, fresh foods, and mentioned that some suppliers also have a very fresh offer of fish and meat (which one parent stated to be better than the supply of fish and meat in the grocery store).

*“It is mainly the local aspect of provider Y that appeals to us rather than the sustainability aspect. During the Corona period, we became more aware of buying local products.” Female, 42 yrs, 13–18 age group*.

*“This is why I chos to switch to provider Z. (…). It really is fresh, all in paper bags, not plastic. So that definitely plays a role for me and it’s also sufficient in terms of portions.” Female, 42 yrs, 6–12 age group*.

### Perceived impact of home-delivered meal boxes

Often the reasons to start using a meal box are linked to their perceived impact. Participants mentioned enhanced cooking knowledge, skills and eating patterns, which are discussed below.

#### Enhanced knowledge and skills

Many participants were enthusiastic about learning new flavor combinations and dishes. This freshly acquired knowledge, in combination with learning new cooking skills, sometimes even led them to abandon their traditional way of cooking (i.e., with potatoes, vegetables and meat). They ate more varied (i.e., by trying other vegetables than they usually ate), and used different cooking methods such as preparing vegetables in the oven, instead of steaming or boiling. Some participants mentioned enjoying stepping out of their comfort zone and leaving traditional routines behind.

*“I learned a lot, also about seasoning. Yes, I found that very enriching, and also working with other products like lentils and beans. I never used to work with that until I started serving it at home since a couple of years” Female, 35 yrs, 6–12 age group*.

*“But mostly I got rid of the pattern of having to eat minced meat, or a derivate of it, almost every day. On that level, it is a breath of fresh air because you get to know a lot of new things and also a lot of spices etc. And indeed, you can now start making combinations yourself, that you don’t find in the boxes, for example, but that you have learned to be inventive with.” Female, 51 yrs, 13–18 age group*.

*“The way of cooking, for example, the vegetables in the oven instead of boiling because you lose much less vitamins, that was something new. If we don’t have the meal box, we still do it. Our way of cooking has changed because of it.” Female, /, 13–18 age group*.

This new way of cooking seemed to be a newly acquired skill, even without the continued use of meal boxes. A few parents with younger children kept the preferred menus to re-prepare them later. Also, some parents from both age categories mentioned experimenting with the recipes or food surpluses to adapt them to their taste.

*“I still use those tricks now, when I make a regular spaghetti bolognaise I put half minced meat and half lentils and they still don’t taste it. It’s just tricks to eat less meat, but still have the protein.” Female, 35 yrs, 6–12 age group*.

Only participants with adolescent children stated that some of them showed interest in cooking together or alone. Because a recipe manual was provided, other members of the family (i.e., children and partners) could easily learn how to prepare a dish.

*“The daughters also cooked themselves or helped out. Yes, that was actually a social event with us. It was cozy.” Female, /, 13–18 age group*.

*“I have made things myself that I had thought I was never going to be able to do, I panic faster than my wife and I am quite a bit stressy.” Male, 38 yrs, 6–12 age group*.

#### Healthier and more varied eating patterns

Almost all participants in both groups perceived meal boxes as healthy. Even though not all participants consciously chose meal boxes because of their healthiness, the positive impact was still clear according to the participants. Most participants stated that their meals were more varied (e.g. every week a mix of fish, meat and vegetarian dishes), that the portion size was adapted to their needs (i.e., not too much), and that they ate less take away and more vegetables. Some participants specifically chose the meal boxes because of these positive effects. Also, the new cooking skills they learned were healthier and more sustainable, such as using Greek yogurt instead of cream to make a sauce or using lentils instead of minced meat.

*“Yes, it does help to be healthier because otherwise we easily reach for take away.” Male, 38 yrs, 6–12 age group*.

*“With provider X you do notice that the vast majority are vegetables and that you do eat healthier. I do pay attention to it, that when you don’t use the meal box anymore, you still make more vegetables.” Female, 40 yrs, 6–12 age group*.

*“With meal boxes you think “hmm it’s gone, I would have eaten some more” but that doesn’t mean you are hungry! And you end up having more than enough. It’s just adjusting your way of eating a little bit.” Female, 51 yrs, 13–18 age group*.

#### More plant-based meals

Many participants in both age groups mentioned eating more vegetarian dishes than before using the meal boxes, or at least learned to appreciate them. They reported having a different, more negative perception of dishes without meat, which changed into being pleasantly surprised after trying them. Nevertheless, a few participants with children in the younger age category stated not choosing these dishes, since they or their family did not like them.

*“In the beginning, I thought this was going to be very difficult with my family as they all like to eat meat, but in the end, nobody said: “There is no meat in it”. On the contrary they are surprised.” Female, 51 yrs, 13–18 age group*.

*“I used to have the wrong idea about vegetarian food too. But with provider X I’ve also taken some vegetarian dishes and actually it’s like candidate 12 says too, I’ve learned to eat that too and it’s even tasty.” Female, 40 yrs, 6–12 age group*.

*“We always pretty traditionally stick to things we know and not the painful choices (ref. to vegetarian).” Male, 38 yrs, 6–12 age group*.

## Discussion

This study explored the determinants and perceived impact of home-delivered meal box use on family meal practices. Parents chose meal boxes because of practical reasons such as saving time and money, and because of the inspiration they provide to cook a fresh meal. Moreover, the tastiness and menu variation were important determinants in choosing a meal box. Meal box providers were compared and chosen based on environmental impact, the freshness and quality of the products, and the price. When looking at the impact of home-delivered meal boxes on meal practices, most parents mentioned enhanced cooking skills and knowledge. Also, attitudes regarding more special or vegetarian dishes changed positively. Lastly, parents stated that their family ate healthier and more often vegetarian. While many results were similar for parents with children in both age categories, there were some differences in the determinants of and the impact of the use of home-delivered meal boxes. In what follows, the results will be discussed more thoroughly.

Regarding parents’ motivations to choose a home-delivered meal box, the four types of motivations of the Motivated Consumer Innovativeness Scale [23] were mentioned, namely: functional (e.g., convenience), hedonic (e.g., tasty recipes), social (e.g., cook together with children), and cognitive (e.g., developing cooking skills, receiving inspiration). Parents mostly choose home-delivered meal boxes because of the practical support they provide in the planning and preparing of meals. Research shows that consumers often seek more convenience (i.e., functional motivation) in their busy daily lives, which they can find in ready-to-cook meals (e.g., home-delivered meal boxes) [14, 15, 24]. Almost all parents also mentioned the importance of learning new cooking techniques and taste combinations (i.e., hedonic and cognitive motivations). As they applied the new ways of cooking even without the continued use of meal boxes, they seemed to have acquired these skills. This finding is in line with prior research that showed significant increases in cooking self-efficacy and cooking techniques following meal box use [25]. The effect seemed to be passed on to the whole family as well, with some adolescent children showing interest in cooking the meals. Older children’s involvement in the cooking and eating of home-delivered meals was confirmed by Utter and colleagues [13], where adolescents enjoyed the process and appreciated the shared moment with their caregivers (i.e., social motivation). In turn, the perceived effects of meal boxes can become motives to use them further. Moreover, parents mentioned that they experienced a reduced mental load by being guided in their food-related decisions and receiving the necessary inspiration for fresh meals (i.e., cognitive motivation), as confirmed by Fraser and colleagues [14]. The importance of this cognitive work is also pointed out in the Family Meal Framework [11], which represents the core components of a family meal (i.e., cognitions, actions, outcomes, beliefs and feelings, and responsibilities). A meal box might support this cognitive load and the actions needed to take, resulting in reduced effort and time.

Our study showed positive effects on various food literacy aspects among parents, as well as among adolescent children. Food literacy describes the set of skills, knowledge and behaviors needed to navigate the food system, by identifying four components: (1) planning and management, (2) selection, (3) preparation, (4) eating [26]. Results showed positive effects on the planning and selection of foods due to home-delivered meal boxes, by enhancing convenience and supporting food-related decisions. Also, as stated earlier, older children in our study were often involved in the planning, selection and preparation of the meals, while younger children were less or not involved. However, research shows that children’s early involvement in food-related tasks could encourage them to try various foods [27]. The home food environment, of which parents are the key gatekeepers, is a crucial determinant in children’s dietary behavior [28]. Parents with younger children seemed to be more influenced by their children’s food preferences in selecting the foods, than parents with older children. This might be because parents know that younger children still have evolving taste preferences and palates [29], and therefore want to take these into account when choosing the meals. Looking at the fourth aspect of food literacy, consuming meals, home-delivered meal boxes seem to contribute to healthier diets due to more variation, appropriate portion sizes and eating more vegetables. Most parents also stated having learned to appreciate vegetarian dishes, to the point where they now even include these in their meal planning. Literature confirms enhanced healthy nutrition due to home-delivered meal boxes [12–14, 30], as well as the importance of home-cooked meals on healthy eating in both children and parents [5–7, 31]. While in the older age category, adolescents mostly had a positive influence on sustaining the use of meal boxes and even suggesting to choose vegetarian meals, in the younger age category, children were sometimes the reason parents stopped using meal boxes. However, still some parents reported convincing the children to try the food or preparing the meals more child-friendly. We might therefore conclude that meal boxes can facilitate families with children to taste new foods and eat more varied.

Our study adds to the literature by considering the role of children and adolescents in the decision to use a meal box and in the perceived impact of home-delivered meal boxes. So, to reach families to improve their meal practices, it is vital to consider influences of children of different ages. Meal box providers could reflect on how the product can be optimized for families with younger and older children, and health professionals could think about how younger children can be engaged more in the preparation of the meals. For example, different preparation techniques (suitable for a wide range of age categories) could be included in the menus. Further research into the role of younger and older children in meal box use, using a larger sample size is warranted to explore different effects by age. Another important finding of our study was the acceptance and consumption of plant-based meals as an effect of meal box use, which should be investigated further in a controlled study to be able to quantify and demonstrate the statistical significance of this perceived effect.

The qualitative evidence provided is essential to gain a deeper understanding of the context surrounding meal box use in families. Despite our study’s strengths, it also has some limitations. First, the study was coordinated by the authors MV and WVL but conducted by two research assistants. Research assistants received a handbook and a short training on how to conduct focus groups. The first author ensured the quality of the data by performing an independent analysis, and excluding some answers on more steering questions. Second, the focus groups were conducted digitally, which can pose a challenge building rapport with participants compared to face-to-face focus group interviews [32]. However, online focus groups were the only possibility to collect data during the Covid-19 restrictions. These video interviews also have other practical advantages such as reducing barriers of time and distance, thus being able to include participants from a broad geographical region. Also, the Covid-19 period might have influenced the results, in the sense that people could appreciate the home delivery even more. Third, both parents who use home-delivered meal boxes, and those who use fresh packages in grocery stores have been included in the study. Fresh packages in grocery stores have the same features as a meal box, except that they are not delivered at home. Hence, for three participants who used the fresh packages, their experiences might have been slightly different, which is important to keep in mind when interpreting the results. However, also the participants using fresh packages mentioned convenience and inspiration as main determinants. Lastly, it might have been helpful to use a particular framework or theory to analyze and code the data. Now an inductive approach has been used, which allows for clear links between the research objectives and the findings derived from the data, but offers a bit less guidance in the analysis and coding process [19].

## Conclusion

First, this paper provided more insight into the drivers of meal box use. Convenience, inspiration, tastiness and menu variation were specifically mentioned as determinants to start and keep using a meal box. Also, environmental aspects such as sustainable packaging, the use of seasonal products, and the use of locally produced fresh foods seemed to be important to customers. Meal box providers could take these aspects into account when developing their products. Second, home-delivered meal boxes might provide a unique opportunity to enhance families’ meal practices. Most parents experience less stress due to meal boxes’ practical support, report better cooking skills, family bonding moments, more healthy diets and acceptance of plant-based meals. The various positive effects on families’ meal practices and the insights in the determinants of meal box use, provide health promotors and social marketeers with crucial information to adopt an optimal strategy to reach families.

### Electronic supplementary material

Below is the link to the electronic supplementary material.

Supplementary Material 1

Supplementary Material 2

## Data Availability

The datasets generated during the current study are not publicly available due to privacy reasons, but are available from the corresponding author on reasonable request.
