# Supplementary material for: Home-delivered meal boxes in a family setting: a qualitative study investigating reasons for use and perceived impact on meal practices
Source: BMC Public Health. 2024 Jan 23;24:277. doi: 10.1186/s12889-024-17729-1 (PMC10807195; doi:10.1186/s12889-024-17729-1)
Supplement: Supplementary file 1 — Supplementary Material 1 [file 12889_2024_17729_MOESM1_ESM.docx]

**Additional file 1.** Interview guide

| QUESTIONS | POINTS OF ATTENTION | | |
| --- | --- | --- | --- |
| *Introduction + opening question* | | | |
| Welcome everyone and thank you for participating in this focus group. I am xxx and this is my colleague xxx and she will take note of the conversation. We are here today to exchange experiences about using meal boxes and fresh food packages. There are no right or wrong answers. Even if others have a different opinion, feel free to express your opinion as well. To make this easier for everyone, there are some agreements we make:  - The information given here stays within this group.  - You have the right to stop your participation in the focus group at any time, and if you do not wish to talk about something you may indicate that.  - You may speak freely, you don't have to raise your hand.  - We do not interrupt participants  **You remain anonymous throughout the discussion and further research. The focus group will be recorded. The recording will be deleted after the research and will only be listened to by the research team. Does everyone agree to this? The focus group will take about an hour.**   1. You may briefly introduce yourself and tell whether or not you like to cook. | - **Informed consent should be signed by every participant** - **Audio recording has started** - **Name, family situation, cooking experience** | | |
| *Transition questions* | | |  |
| 1. What was the reason you decided to use meal boxes? 2. In your opinion, is the choice offer sufficient? Or are certain needs not being met? | | - **Open question - leaving room for silence**   - Healthier lifestyle?   - Sustainability (reduce food waste)?   - Convenience - saving time?   - Price-quality correct? - **What do you think is still missing?** - **Continued use of meal boxes?** | |
| 1. How important do you feel it is to maintain a healthy lifestyle? | | - **Does a meal box help with this?**   - Do you think meal boxes are healthier than preparing your own meals?   - Do you think meal boxes are healthier than other options within convenience foods such as ready-to-eat meals? | |
| *Key questions* | | | |
| 1. In what ways and to what extent do you think consuming a meal box has changed your eating behaviors/customs? 2. What do you think are the advantages of a meal box? 3. What do you think are the disadvantages of a meal box? 4. What role do your children play in choosing meal boxes and/or fresh packages? | | - What habits have changed?   - Cooking skills?   - Inspiration (recipes)?   - Vegetarian? More varied? - Food waste? - Saving time? - Does this encourage eating vegetables/new flavors? - Does this also encourage eating vegetarian more often? | |
| *Final questions* | | | |
| 1. Of all the things we discussed, what is most important to you? What do you remember most? 2. Overall, how satisfied are you about meal boxes? 3. To what extent do you think your children are influenced by the use of meal boxes and/or fresh packages? | | - Which aspect has the greatest impact/is most important to them? | |
| 1. Is this a good summary of the conversation? | | - **The conversation is summarised to check whether the interpretation is correct.** | |
| 1. Is there anything else you want to say about the topic, that we haven't talked about yet? | |  | |
