# Supplementary material for: Home-delivered meal boxes in a family setting: a qualitative study investigating reasons for use and perceived impact on meal practices
Source: BMC Public Health. 2024 Jan 23;24:277. doi: 10.1186/s12889-024-17729-1 (PMC10807195; doi:10.1186/s12889-024-17729-1)

**Additional file 2.** Demographic survey

**Below we ask some questions about you and your family.**

**Date: ...**

1. You are

Man
Woman

1. What is your age?

…

1. How many children do you have (including stepchildren, etc.)?

…

1. How old are your child(ren)?

…

1. How many children eat with you when you prepare a meal from a meal box/fresh package?

….

1. What is your marital status?

Single

Married

Living together, not married

LAT-relation

Divorced

Widow

Other:_________________

1. What is your **highest school degree** (please do not indicate more than 1 degree)?

No degree

Primary education

Primary education

Special primary education

Lower secondary education (until 3^th^ year):

Vocational education (BSO)

Technical education (TSO)

Art education (KSO)

General education (ASO)

Special education (BuSO)

Higher secondary education (until 6^th^ year):

Vocational education (BSO)

Technical education (TSO)

Art education (KSO)

General education (ASO)

Higher education: type:

Graduate school short type (max. 3 yrs)

Graduate school long type (min. 4 yrs)

University

1. What is your **profession**? Describe your current job. If you have a partner, fill in your partner’s profession.

| **Own profession** | **Partner’s profession** |
| --- | --- |
| Blue collar worker  Skilled worker  Unskilled worker  White collar worker  Lower (eg. nursing staff, administrative assistant, police, …)  Higher (eg. middle management jobs, staff memberr, …)  Management team  Working in education  Primary or secondary education  Higher education or university  Other: …  Self-employed:  Farmer  Self-employed smaller business  ☐ Business leader  ☐ Wholesaler  Independent professionals  Never worked  Unemployed | - Not applicable   Blue collar worker  Skilled worker  Unskilled worker  White collar worker  Lower (eg. nursing staff, administrative assistant, police, …)  Higher (eg. middle management jobs, staff memberr, …)  Management team  Working in education  Primary or secondary education  Higher education or university  Other: …  Self-employed:  Farmer  Self-employed smaller business  ☐ Business leader  ☐ Wholesaler  Independent professionals  Never worked  Unemployed |

1. In the family, who is responsible for food purchases?

Me

My partner

My children

Other: …

1. In the family, who is responsible for cooking dinner?

Me

My partner

My children

Other: …

1. With which of the following groups do you most identify with?
   - Omnivore
   - Flexitarian
   - Vegetarian
   - Vegan
2. What types of convenience foods do you use?

- Meal boxes in subscription system
- Fresh packages from the supermarket
- Both

1. Who is your meal box supplier?

- HelloFresh
- Foodbag
- Marley Spoon
- Ekomenu
- 15gram Foodbox
- Other: …

1. How did you come to this supplier?

- Through recommendation of friends/family/acquaintance
- Through advertising (online, TV,..)
- Through a free test box
- Other: …

1. How long have you been using meal boxes?

….

1. How often do you consume a meal box per week?

- Once a week
- Twice a week
- 3 times a week
- More than 3 times a week

1. If you use meal boxes, how frequently do you order them?
   - Only once per week
   - Only a few months and then stopped
   - For several months now
   - One year or more now
   - In periods
2. If you use meal boxes, how frequently do you purchase them?

- Several times a week
- Only once a week
- Several times a month
- Only once a month
- Less than above frequencies

1. For how many people do you purchase a meal box?

- 1 person
- 2 persons
- 3 persons
- 4 persons
- more than 4 persons

1. How varied are the meals in the longer term?

Not varied Very varied


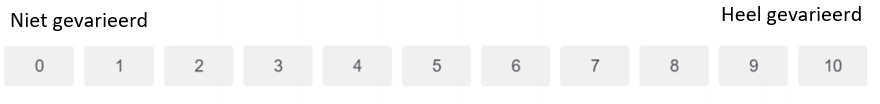


1. How fresh are the ingredients provided in the meal boxes?

Not fresh Very fresh


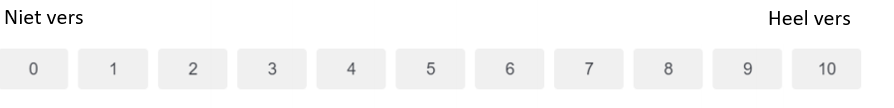


1. In general, how tasty do you find the dishes from meal boxes?

Not tasty Very tasty


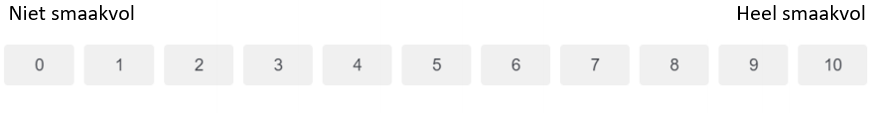


1. Would you recommend meal boxes to your family or friends?

Not likely Very likely


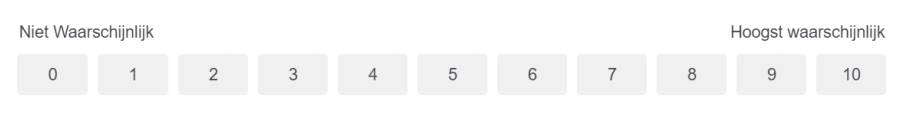

Supplement: Supplementary file 2 — Supplementary Material 2 [file 12889_2024_17729_MOESM2_ESM.docx]
